# Association between triglyceride glucose-body mass index and gestational diabetes mellitus: a prospective cohort study

**DOI:** 10.1186/s12884-025-07294-9

**Published:** 2025-02-17

**Authors:** Xiaomin Liang, Kai Lai, Xiaohong Li, Di Ren, Shuiqing Gui, Ying Li, Zemao Xing

**Affiliations:** Department of Critical Care Medicine, Shenzhen Second People’s Hospital, The First Affiliated Hospital of Shenzhen University, Shenzhen, China

**Keywords:** Triglyceride glucose-body mass index, Gestational diabetes mellitus, Cohort study

## Abstract

**Background:**

Limited research has examined the potential association between triglyceride glucose-body mass index (TyG-BMI) and gestational diabetes mellitus (GDM). The objective of this investigation was to analyze this linkage and evaluate TyG-BMI’s capability to predict GDM.

**Methods:**

This research employed secondary data derived from a prospective cohort in South Korea, which included 588 pregnant women with singleton gestations, collected between November 2014 and July 2016. To investigate the connection between TyG-BMI and GDM, logistic regression and sensitivity analyses were performed. Furthermore, an analysis of receiver operating characteristics (ROC) was conducted to assess the prognostic accuracy of TyG-BMI in relation to GDM.

**Results:**

The cohort exhibited a mean age of 32.07 ± 3.80 years, with 36 individuals (6.12%) manifesting GDM during the interval of 24 to 28 weeks of gestation. Following the adjustment for possible confounding variables, an increased TyG-BMI was associated with an elevated risk of GDM, as indicated by an odds ratio (OR) of 1.02 (95% CI: 1.01–1.04). Additionally, the area under the curve (AUC) for TyG-BMI’s predictive performance was recorded at 0.7979 (0.7143–0.8814), with an optimal threshold established at 211.03, which resulted in a specificity of 86.23% and a sensitivity of 66.67%.

**Conclusions:**

In this South Korean cohort, increased TyG-BMI during early pregnancy (10–14 weeks) was significantly associated with the onset of GDM (during pregnancy 24–28 weeks). TyG-BMI could be integrated into clinical practice as a complementary preliminary screening tool for detecting women who are at increased risk of GDM.

**Supplementary Information:**

The online version contains supplementary material available at 10.1186/s12884-025-07294-9.

## Introduction

Gestational diabetes mellitus (GDM), characterized by varying glucose tolerance levels during pregnancy [1], is increasingly prevalent worldwide, influenced by factors such as rising maternal age and obesity rates, alongside improvements in diagnostic criteria and methods [2]. GDM is the most common pregnancy-related medical condition, affecting about 15% of all pregnancies globally, corresponding to roughly 18 million births annually [3]. This condition presents both acute and chronic health risks for mothers and their offspring [4–6]. GDM is generally diagnosed between the 24th and 28th weeks of gestation [7]. At this point, pregnant women and their fetuses may already be at increased risk for negative outcomes and enduring endocrine issues due to early glucotoxic effects [8, 9]. Early identification of individuals at elevated risk for GDM is therefore essential to minimize adverse impacts and prevent transgenerational metabolic diseases.

Insulin resistance (IR) plays a pivotal role in the etiology of GDM, but identifying the risk for GDM at an early stage remains a significant challenge in clinical settings [10]. While the euglycemic hyperinsulinemic clamp serves as the gold standard for assessing insulin sensitivity, its use in everyday clinical settings is limited due to high costs, labor demands, and intricate technical requirements [11–14]. Alternative predictive methods based on single parameters, such as Body Mass Index (BMI), Triglyceride (TG), or Fasting Plasma Glucose (FPG), have proven to be inadequately accurate [14, 15]. Considering the intricate pathophysiology of GDM, these individual markers do not adequately reflect its multifactorial nature, underscoring the necessity for more comprehensive predictive models that incorporate multiple risk factors [16–18].

The Triglyceride Glucose-Body Mass Index (TyG-BMI) serves as a sophisticated metabolic indicator, integrating various components: TG, FPG, and BMI. The TyG-BMI index demonstrates a robust correlation with the homeostasis model assessment of insulin resistance (HOMA-IR) and exceeds conventional metrics in predictive accuracy [19, 20]. In addition to its function in assessing IR, TyG-BMI is associated with various metabolic disorders, such as nonalcoholic fatty liver disease (NAFLD), hypertension, and diabetes [21–24]. Its holistic approach allows for a more precise detection of metabolic disturbances, particularly in identifying abnormalities in dyslipidemia and glucose homeostasis [25, 26].

Despite TyG-BMI’s acknowledged value in assessing metabolic risk, its association with GDM during early gestation remains underexplored. Our study, a prospective cohort analysis, was undertaken to investigate TyG-BMI’s specific correlation with GDM and to assess its predictive utility for GDM from the 10th to the 14th week of gestation—well ahead of the typical screening timeline (24–28 weeks). By comparing it with established metabolic markers and identifying optimal cutoff values, this study aims to facilitate early assessment of GDM risk.

## Methods

### Study design

This analysis was conducted using a prospective cohort study design. A team of researchers in South Korea established the “Fatty Liver in Pregnancy” registry (Clinical Trial Number: NCT02276144, Clinical Trial Registry: https://www.clinicaltrials.gov/, Registration Date: October 28, 2014) as the source of the data utilized in this study. The dataset was derived from an established trial database [27]. Between November 2014 and July 2016, the cohort of pregnant women receiving prenatal care at the Seoul Metropolitan Government Seoul National University Boramae Medical Center and Incheon Seoul Women’s Hospital was identified as singletons. The objective of the study was to investigate the impacts of NAFLD.

### Data source

An earlier study by Lee et al. provided the main data used in this investigation [28]. Under the Creative Commons Attribution License, you can copy, distribute, and use it as you like as long as you provide credit where credit is due. Our deepest appreciation goes out to everyone who put in the time and effort to provide us with the data.

### Study population

The initial research received approval from the Public Institutional Review Board of the Korean Ministry of Health and Welfare, as well as the Institutional Review Board of the Seoul Metropolitan Government Seoul National University Boramae Medical Center. Each participant was fully informed about the study’s purpose, procedures, and potential risks and benefits, and provided written informed consent before enrollment. Furthermore, both the initial and secondary analyses followed the rules set out by STROBE, and were compliant with the Declaration of Helsinki [29]. All steps of this study were compliant with all applicable laws and standards.

**Fig. 1:**
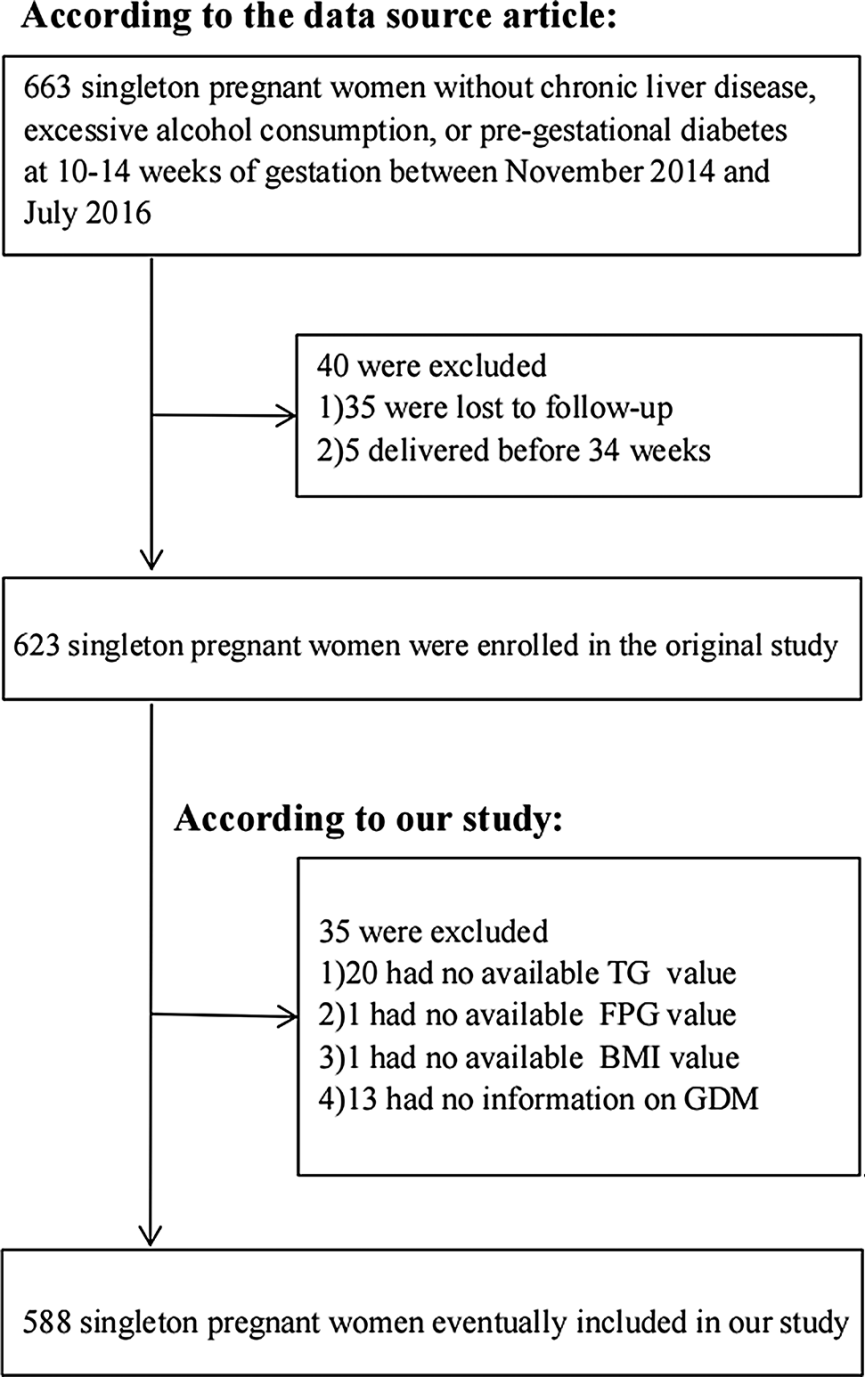
Flowchart of study participants

In initial study, 663 singleton women without pregestational diabetes, heavy alcohol consumption, or chronic liver disease were enrolled. There were 623 women who could still take part in the study after 40 were removed for follow-up loss or for being born preterm (before 34 weeks). Thirteen participants were eliminated from the secondary analysis owing to missing data on GDM, and twenty-two were omitted because of exposure variables (TG, FPG, and BMI). Figure 1 shows the final study population of 588 women.

### Data collection

The data was compiled and organized by trained medical experts. For the purpose of collecting broad demographic and clinical data (parity, diabetes history, alcohol consumption, pre-pregnancy BMI, and age), a standardized survey was utilized. Hematological indicators were assessed by drawing venous blood samples at 10–14 weeks of gestation following an 8-hour fast, including insulin levels, alanine aminotransferase (ALT), gamma-glutamyl transferase (GGT), total cholesterol (TC), TG, high-density lipoprotein cholesterol (HDL), low-density lipoprotein cholesterol (LDL), FPG, and aspartate aminotransferase (AST). A semiquantitative scale with grades 0–3 was used to evaluate hepatic steatosis [30]. Standard procedures were used to determine the HOMA-IR [31]. A two-stage GDM screening was administered to participants between 24 and 28 weeks gestation [32, 33].

### Variables

#### TyG-BMI

The TyG-BMI, calculated as noted in $\:ln\:\left[FPG\:\right(mg/dL)\:\times\:\:TG\:(mg/dL)/2]\times\:BMI(kg/m$^*2*^$\:)$ [19], served as the exposure variable.

#### GDM

The International Association of Diabetes and Pregnancy Study Groups [34] developed criteria for the identification of GDM, which involved a two-step technique applied between 24 and 28 weeks of gestation, as described by Lee et al. [28]. To begin, participants were given a 50 g oral glucose challenge test (GCT) while not fasting, and then their blood glucose levels were measured one hour after the test. GCT value of 7.8 mmol/L or greater was considered positive. Those who had a positive result were then given another oral glucose tolerance test (OGTT) of 100 g. The following criteria must be met in order to confirm a diagnosis of GDM: FPG of 5.3 mmol/L or higher, one-hour glucose of 10 mmol/L or higher, two-hour glucose of 8.6 mmol/L or higher, or three-hour glucose of 7.8 mmol/L or higher [28, 34].

#### Missing data handling

Missing data were expressed as a percentage and included ALT (2, 0.34%), AST (2, 0.34%), and insulin (1, 0.17%). An imputation strategy involving multiple imputations was utilized to address missing data and reduce bias, potentially affecting the accuracy and statistical integrity of the findings [35, 36]. This analysis assumed that missing data were missing at random [35].

### Statistical analysis

The data are expressed as the mean ± standard deviation (SD). Frequency distributions were utilized to summarize categorical variables, expressed as percentages. Differences among groups were evaluated through chi-square tests for categorical variables, Kruskal-Wallis tests for variables that do not follow a normal distribution, and one-way ANOVA for those that do exhibit a normal distribution.

Confounding variables were identified based on their relevance to the outcomes studied or their ability to alter the effect estimates by more than 10% [29]. Due to their collinearity, HOMA-IR and TC were excluded from the list of adjusted covariates (Supplementary Table 1). Drawing from a comprehensive review of existing literature, alongside medical expertise and initial investigations into the risk factors associated with GDM, various covariates were identified for inclusion [27, 28, 37]. These covariates encompass age, parity, AST, insulin, LDL, hepatic steatosis, GGT, ALT, and HDL. Logistic regression was utilized to calculate odds ratios (OR) and 95% confidence intervals (CI) to examine the relationship between TyG-BMI and GDM.

A stratified logistic regression approach was utilized to evaluate subgroup consistency, focusing on variables such as hepatic steatosis, HOMA-IR, parity, and age. Likelihood ratio tests were utilized to investigate the interactions between TyG-BMI and GDM across these subgroups. To ensure the reliability of the results, participants with a pre-pregnancy BMI ≥ 25 kg/m^2^ or higher, as well as those with grade 1–3 hepatic steatosis, were excluded from the sensitivity analyses due to the established link between obesity and hepatic steatosis and the increased prevalence of GDM [27, 37]. E-values were calculated to assess the potential impact of unmeasured confounding variables on the relationship between TyG-BMI and GDM [38].

Subsequently, a receiver operating characteristic (ROC) curve was constructed to evaluate the efficacy of TyG-BMI and other markers in predicting GDM, by calculating the area under the curve (AUC) and identifying the optimal threshold.

All statistical methods followed the STROBE guidelines [29]. Statistical analyses were performed utilizing R software (version 4.2.0) and EmpowerStats (version 4.2). The threshold for statistical significance was established at p-values < 0.05.

## Results

### Characteristics of participants

**Table 1:** Characteristics of participants

Characteristics	Overall	TyG-BMI tertile			*P* value
		T1(118.73-166.15)	T2(166.16-190.48)	T3(190.49-338.58)	
Participants	588	196	196	196	
Age (years)	32.07 ± 3.80	31.84 ± 3.68	32.01 ± 3.64	32.35 ± 4.07	0.399
GGT (IU/L)	12.00 (10.00–15.00)	11.00 (9.00–14.00)	11.00 (9.75-15.00)	14.00 (11.00–19.00)	< 0.001
ALT (IU/L)	11.00 (8.00–15.00)	10.00 (8.00–13.00)	11.00 (8.00–15.00)	13.00 (9.00-17.50)	< 0.001
AST (IU/L)	16.00 (14.00–20.00)	16.00 (14.00–19.00)	16.00 (14.00–20.00)	16.00 (14.00–20.00)	0.531
Insulin (µIU/mL)	8.40 (5.40-11.55)	6.35 (4.07–9.03)	8.40 (5.35–11.20)	11.00 (7.47–16.42)	< 0.001
Pre-pregnancy BMI (kg/m^2^)	22.02 ± 3.48	18.94 ± 1.29	21.39 ± 1.21	25.73 ± 3.11	< 0.001
TC (mg/dL)	172.80 ± 27.21	163.59 ± 23.61	171.47 ± 26.12	183.34 ± 28.11	< 0.001
TG (mg/dL)	118.82 ± 47.49	94.18 ± 28.53	116.77 ± 38.59	145.51 ± 56.18	< 0.001
HDL (mg/dL)	64.90 ± 13.55	66.54 ± 13.03	64.12 ± 13.43	64.04 ± 14.10	0.115
LDL (mg/dL)	84.02 ± 21.81	78.23 ± 19.39	83.62 ± 20.48	90.20 ± 23.74	< 0.001
FPG (mg/dL)	77.01 ± 9.72	74.62 ± 8.70	76.97 ± 8.24	79.42 ± 11.36	< 0.001
HOMA-IR	1.50 (1.00-2.30)	1.10 (0.70–1.70)	1.60 (1.00-2.20)	2.20 (1.30–3.12)	< 0.001
Parity					0.030
No	309 (52.55%)	109 (55.61%)	112 (57.14%)	88 (44.90%)	
Yes	279 (47.45%)	87 (44.39%)	84 (42.86%)	108 (55.10%)	
GDM					< 0.001
No	552 (93.88%)	193 (98.47%)	189 (96.43%)	170 (86.73%)	
Yes	36 (6.12%)	3 (1.53%)	7 (3.57%)	26 (13.27%)	
Hepatic steatosis					< 0.001
Grade 0	478 (81.29%)	182 (92.86%)	170 (86.73%)	126 (64.29%)	
Grade 1	85 (14.46%)	14 (7.14%)	24 (12.24%)	47 (23.98%)	
Grade 2	17 (2.89%)	0 (0.00%)	1 (0.51%)	16 (8.16%)	
Grade 3	8 (1.36%)	0 (0.00%)	1 (0.51%)	7 (3.57%)	

Values are reported as mean ± SD, median (Q1-Q3), or N (%)

The study population comprised 588 eligible pregnant women, with a mean age of 32.07 ± 3.80 years. A total of 36 women, representing 6.12%, received a diagnosis of GDM during the period of 24 to 28 weeks of gestation. Table 1 presents the baseline characteristics of the participants, categorized by TyG-BMI tertiles. Participants in the highest TyG-BMI tertile (T3) exhibited elevated levels of FPG, HOMA-IR, TC, GGT, ALT, insulin, BMI, TG, and LDL, alongside increased risks of GDM and hepatic steatosis (Table 1).

### Association between TyG-BMI and GDM

**Table 2:** Results of univariate analysis

	Statistics	OR (95%CI)	*P* value
Age (years)	32.07 ± 3.80	1.04 (0.95, 1.13)	0.4253
Pre-pregnancy BMI (kg/m^2^)	22.02 ± 3.48	1.28 (1.18, 1.38)	< 0.0001
Insulin (µIU/mL)	8.40 (5.40-11.55)	1.12 (1.07, 1.17)	< 0.0001
GGT (IU/L)	12.00 (10.00–15.00)	1.03 (1.01, 1.06)	0.0115
ALT (IU/L)	11.00 (8.00–15.00)	1.04 (1.01, 1.06)	0.0018
AST (IU/L)	16.00 (14.00–20.00)	1.02 (0.99, 1.05)	0.1739
Parity			
No	309 (52.55%)	1.0	
Yes	279 (47.45%)	0.99 (0.50, 1.95)	0.9776
FPG (mg/dL)	77.01 ± 9.72	1.07 (1.04, 1.10)	< 0.0001
LDL (mg/dL)	84.02 ± 21.81	1.00 (0.98, 1.02)	0.9984
HDL (mg/dL)	64.90 ± 13.55	0.96 (0.94, 0.99)	0.0061
TG (mg/dL)	118.82 ± 47.49	1.02 (1.01, 1.02)	< 0.0001
TC (mg/dL)	172.80 ± 27.21	1.01 (1.00, 1.02)	0.0921
HOMA-IR	1.50 (1.00-2.30)	1.48 (1.22, 1.78)	< 0.0001
Hepatic steatosis			
Grade 0	478 (81.29%)	1.0	
Grade 1	85 (14.46%)	3.42 (1.46, 8.02)	0.0047
Grade 2	17 (2.89%)	25.67 (8.76, 75.20)	< 0.0001
Grade 3	8 (1.36%)	17.32 (3.81, 78.87)	0.0002
TyG-BMI	184.25 ± 32.97	1.03 (1.02, 1.04)	< 0.0001

Values are reported as mean ± SD, median (Q1-Q3), or N (%)

Univariate analyses revealed that elevated levels of HOMA-IR, pre-pregnancy BMI, TG, FPG, insulin, GGT, ALT, and grades of hepatic steatosis were significantly associated with an increased GDM risk, while HDL showed an inverse relationship (Table 2).

**Table 3:** Relationship between TyG-BMI and GDM risk in different models

Exposure	Crude model (OR, 95%CI, *P*)	Model I (OR, 95%CI, *P*)	Model II (OR, 95%CI, *P*)	Model III (OR, 95%CI, *P*)
TyG-BMI	1.03 (1.02, 1.04) < 0.0001	1.03 (1.02, 1.04) < 0.0001	1.03 (1.02, 1.04) < 0.0001	1.02 (1.01, 1.04) < 0.0001
TyG-BMI tertile				
T1	1.0	1.0	1.0	1.0
T2	2.38 (0.61, 9.35) 0.2132	2.36 (0.60, 9.26) 0.2189	2.00 (0.50, 7.96) 0.3273	1.77 (0.44, 7.12) 0.4187
T3	9.84 (2.93, 33.07) 0.0002	10.00 (2.96, 33.74) 0.0002	5.46 (1.51, 19.75) 0.0096	3.73 (0.99, 14.12) 0.0522
P for trend	< 0.0001	< 0.0001	0.0035	0.0322

Crude model: unadjusted

Model I: Adjusted for age and parity

Model II: Adjusted for age, parity, AST, LDL, hepatic steatosis, GGT, ALT, and HDL

Model III: Adjusted for age, parity, AST, insulin, LDL, hepatic steatosis, GGT, ALT, and HDL

Table 3 presents a clear and significant correlation between increased TyG-BMI levels and the heightened risk of GDM across all analytical models, regardless of confounder adjustments (Crude model: OR = 1.03 (95% CI: 1.02, 1.04); Model I: OR = 1.03 (95% CI: 1.02, 1.04); Model II: OR = 1.03 (95% CI: 1.02, 1.04); Model III: OR = 1.02 (95% CI: 1.01, 1.04)). Interestingly, Model III, after accounting for various influencing factors, demonstrated a 2% elevation in the risk of GDM with each unit increase in TyG-BMI. A notable pattern of increasing GDM risk associated with higher TyG-BMI tertiles was observed in all models (P for trend < 0.05).

### Sensitivity analysis

**Table 4:** Relationship between TyG-BMI and GDM risk in different sensitivity analyses

Exposure	Model I (OR, 95%CI, *P*)	Model II (OR, 95%CI, *P*)
TyG-BMI	1.03 (1.00, 1.06) 0.0375	1.02 (1.00, 1.04) 0.0120
TyG-BMI tertile		
T1	1.0	1.0
T2	1.83 (0.45, 7.42) 0.3960	1.74 (0.41, 7.43) 0.4522
T3	2.66 (0.59, 12.01) 0.2033	3.90 (1.00, 15.14) 0.0494
P for trend	0.2000	0.0403

Model I adjusted for age, parity, LDL, insulin, AST, ALT, GGT, hepatic steatosis, and HDL in participants with a pre-pregnancy BMI < 25 kg/m^2^ (*n* = 493)

Model II included individuals with grade 0 hepatic steatosis (*n* = 478), adjusting for age, parity, insulin, LDL, ALT, GGT, AST, and HDL

Table 4 details the sensitivity analysis focusing on individuals with a pre-pregnancy BMI < 25 kg/m^2^, confirming a robust association between TyG-BMI and GDM risk (OR = 1.03; 95% CI: 1.00, 1.06). In subjects with grade 0 hepatic steatosis, TyG-BMI also remained significantly linked to GDM risk (OR = 1.02; 95% CI: 1.00, 1.04). These analyses further validate the consistency of our findings.

**Table 5:** Subgroup analysis of the association between TyG-BMI and GDM risk

Subgroup	No of patients	Adjusted OR (95%Cl)	*P* value	*P* for interaction
Age (years)				0.6491
<35	451	1.03 (1.01, 1.04)	0.0001	
≥35	137	1.04 (0.99, 1.08)	0.1052	
HOMA-IR				0.3978
≤2	388	1.02 (1.00, 1.05)	0.0255	
>2	200	1.02 (1.01, 1.04)	0.0038	
Parity				0.4338
No	309	1.03 (1.01, 1.05)	0.0033	
Yes	279	1.02 (1.00, 1.04)	0.0133	
Hepatic steatosis				0.3383
Grade 0	478	1.02 (1.00, 1.04)	0.1040	
Grade 1–3	110	1.03 (1.01, 1.05)	0.0058	

Models were adjusted for age, parity, AST, insulin, LDL, hepatic steatosis, GGT, ALT, and HDL, without adjustment for the stratification variable.

Subgroup analyses were conducted to explore potential modifiers in the relationship between TyG-BMI and the risk of GDM, emphasizing age, HOMA-IR, parity, and hepatic steatosis as stratification factors (Table 5). The analyses indicated that the previously mentioned confounders did not have a significant impact on the association between TyG-BMI and the risk of GDM.

The E-value was calculated to determine the minimum strength of association an unmeasured confounder would require with both the exposure and outcome to negate the observed relationship, indicating that unmeasured confounding is improbable to substantially impact the validity of our results.

### ROC analysis

**Table 6:** AUC for each assessed parameter in determining GDM

Variables	AUC (95% CI)	Best threshold	Specificity	Sensitivity	Youden index
TyG-BMI	0.7979(0.7143–0.8814)	211.0316	0.8623	0.6667	0.529
BMI (kg/m^2^)	0.7477(0.6538–0.8415)	23.6700	0.7808	0.6667	0.4475
TG (mg/dL)	0.7806(0.7045–0.8566)	121.5000	0.6431	0.8333	0.4764
FPG (mg/dL)	0.6592(0.5553–0.7632)	90.5000	0.9583	0.3056	0.2639
Insulin (µIU/mL)	0.7643(0.6753–0.8534)	13.9000	0.8675	0.6111	0.4786
HOMA-IR	0.7655(0.6795–0.8516)	2.7500	0.8768	0.5833	0.4601

**Fig. 2:**
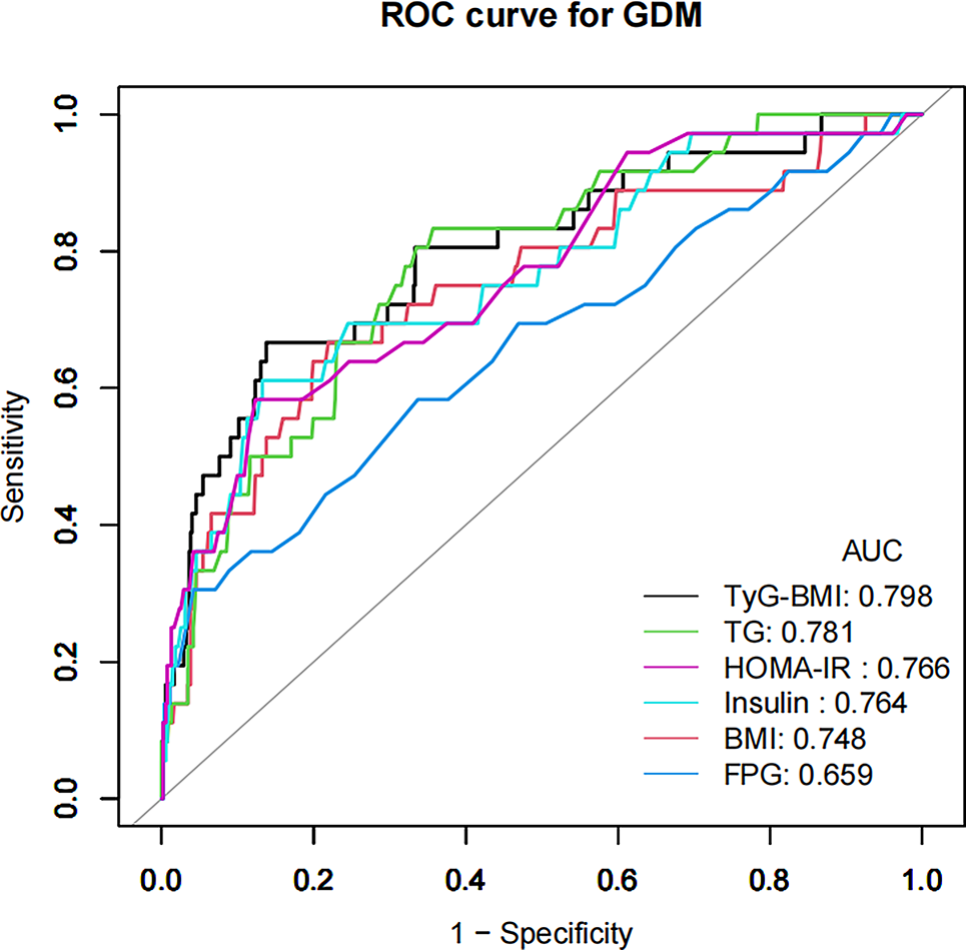
ROC curves for TyG-BMI predicting GDM risk in all subjects. The analysis revealed that TyG-BMI achieved an AUC of 0.798, surpassing TG, BMI, FPG, insulin, and HOMA-IR in predictive accuracy for GDM

The predictive efficacy of TyG-BMI for GDM was assessed using ROC analysis (Table 6; Fig. 2), yielding an AUC of 0.7979 (95% CI: 0.7143–0.8814). Interestingly, TyG-BMI exhibited the highest AUC when compared to other variables including TG, BMI, FPG, insulin, and HOMA-IR, highlighting its enhanced predictive ability for GDM. The optimal TyG-BMI threshold for predicting GDM was identified at 211.03 using Youden’s index, yielding a specificity of 86.23% and a sensitivity of 66.67% (Table 6).

## Discussion

This study examined the relationship between TyG-BMI and the risk of GDM in a cohort of 588 singleton pregnant women from South Korea, demonstrating that TyG-BMI serves as a significant risk factor and shows a positive correlation with GDM incidence. The sensitivity analysis supported this connection. Moreover, TyG-BMI demonstrated a significant ability to predict GDM, attaining an AUC of 0.7979 with an optimal threshold of 211.03, reflecting both elevated specificity and sensitivity. Notably, TyG-BMI demonstrated superior performance compared to traditional indicators like TG, BMI, insulin, FPG, and HOMA-IR in the prediction of GDM, suggesting its potential utility as an early screening instrument in clinical environments for identifying women at increased risk for GDM.

The prevalence of GDM among the South Korean population has increased, reaching 12.70% [39]. In our study, the incidence of GDM was 6.12%, which is lower than the reported rate. This discrepancy could be attributed to the stringent exclusion criteria (pregestational diabetes, heavy alcohol use, and chronic liver illness) and more rigorous GDM diagnostic standards (two-step approach) used in our study, which may have influenced the lower incidence observed. However, the persistence of a 6.12% prevalence underscores the importance of further investigating potential GDM risk factors.

TyG-BMI, integrating both BMI and the TyG index, offers a more comprehensive measure of IR than any single parameter [19]. Meta-analytic evidence from 33 observational studies involving 962,966 women indicated a 4% increase in GDM risk per unit increase in pre-pregnancy BMI [40]. Moreover, overweight or obese pregnant women exhibited a 23% greater risk of developing GDM [41]. Additionally, the TyG index has shown positive correlations with GDM in diverse regions such as Iran, Hungary, Korea, Mexico, and China [42–46]. Moreover, studies demonstrate that higher TyG-BMI indices correlate with a greater likelihood of developing diabetes. In a cohort study, Song et al. observed a notable correlation between TyG-BMI levels and the incidence of diabetes over a 13-year duration within a Japanese cohort (HR = 1.015, 95% CI: 1.011–1.019) [23]. Concurrently, Wang et al. validated its predictive capacity for diabetes among Chinese individuals over a period of 3.1 years (HR 1.50 per SD increase, 95% CI: 1.40–1.60) [24]. Nevertheless, the association between TyG-BMI and GDM remains inadequately explored. Our examination revealed a notable positive association between elevated TyG-BMI levels and the likelihood of developing GDM, indicated by an OR of 1.02 (95% CI: 1.01–1.04). This relationship was consistent across various subgroups, including those differentiated by age, HOMA-IR, parity, and hepatic steatosis, thereby reinforcing the credibility and generalizability of our results.

Song et al. established an optimal TyG-BMI threshold of 197.2987 for predicting diabetes incidence, featuring an AUC of 0.7738 [23]. Similarly, Wang et al. determined a cutoff of 213.2966 for TyG-BMI that effectively forecasts the emergence of diabetes, marked by an AUC of 0.7741, with 69.54% specificity and 72.51% sensitivity [24]. In our research, TyG-BMI exhibited substantial predictive capability for GDM, highlighted by an AUC of 0.7979 and a specific predictive cutoff of 211.0316. Furthermore, TyG-BMI outperformed TG, BMI, HOMA-IR, insulin, and FPG in predicting GDM. Surprisingly, TyG-BMI exhibited higher diagnostic accuracy for GDM than HOMA-IR, highlighting its potential as a reliable indicator of GDM diagnosis and an early biomarker for IR in pregnancy. Therefore, we recommend incorporating TyG-BMI assessments into routine prenatal screenings during early pregnancy. Should the TyG-BMI of a pregnant woman exceed 211.0316 early in gestation, she may be considered at elevated risk for GDM, and preemptive measures might be advisable to mitigate or circumvent the detrimental outcomes historically noted in pregnant women and fetuses before the typical diagnostic window of 24–28 weeks.

Although the precise mechanisms connecting TyG-BMI with GDM remain to be fully defined, substantial evidence supports that this link is influenced by intricate interactions between obesity-related hormones and adipokines [47]. Obesity disrupts adipokine equilibrium, primarily through an increase in leptin levels, which is associated with IR and maternal hyperglycemia [48, 49], and a reduction in adiponectin, leading to hypoadiponectinemia frequently seen in GDM [50, 51]. Such disruptions in adipokine balance foster a pro-inflammatory state that aggravates metabolic dysfunction during pregnancy. Additional adipokines, such as resistin and visfatin, contribute to this process by further affecting insulin sensitivity [52, 53]. Crucially, these metabolic changes impact not only maternal health but also fetal development, possibly increasing the risk of metabolic disorders in offspring [54, 55]. This framework elucidates how TyG-BMI, integrating glycemic status and body adiposity, effectively forecasts GDM risk through these interrelated physiological pathways.

### Study strengths and limitations

This investigation has several distinct advantages. It appears to be the inaugural study examining the connection between TyG-BMI from early pregnancy (10–14 weeks) and GDM in the pregnancy stages (24–28 weeks) within a South Korean cohort. Our methodical prospective cohort design, employing stringent criteria for participant inclusion and exclusion, ensured the orderly collection of data and the derivation of dependable outcomes. Our findings, demonstrating a consistent and significant link between TyG-BMI and GDM, are reinforced even after adjustments for multiple confounders. Sensitivity and subgroup analyses further affirm the robustness of these observations. Our studies suggest that TyG-BMI provides superior predictive accuracy over traditional measures such as TG, BMI, FPG, insulin, and HOMA-IR, with a well-defined optimal threshold. Significantly, assessing TyG-BMI early in pregnancy provides a unique opportunity for early detection and proactive intervention before typical GDM screening.

Nevertheless, our study’s emphasis on a South Korean demographic poses a limitation in terms of the generalizability of these results to other ethnic and metabolic profiles. Further research, encompassing a broader, more varied demographic, BMI, and metabolic profiles, is essential to substantiate the TyG-BMI and GDM association across diverse groups. Additionally, the possibility of unaccounted confounding factors remains, despite thorough adjustments, though our E-value calculations suggest minimal impact on the results. The limitation of a single TyG-BMI measurement between weeks 10–14 does not account for potential gestational fluctuations, highlighting the need for longitudinal studies in multiple centers. Our study’s restriction to singleton pregnancies also suggests the necessity for future work to evaluate the efficacy of TyG-BMI in predicting GDM in multiple gestations, known to present a higher GDM risk [56, 57]. Lastly, the absence of data on conception methods (natural versus assisted reproductive technology), which may influence GDM risk [58], points to an area for future research to explore the implications of TyG-BMI in pregnancies achieved through assisted reproductive technologies.

## Conclusion

In the South Korean population, we identified a robust positive correlation between early pregnancy TyG-BMI (10–14 weeks) and the subsequent diagnosis of GDM in pregnancy stages (24–28 weeks). TyG-BMI could be integrated into clinical practice as a complementary preliminary screening tool for detecting women who are at increased risk of GDM. To extend these results and evaluate their relevance to a more diverse population, forthcoming research should be broader, incorporating varied ethnicities and encompassing extensive age ranges, BMI categories, and metabolic conditions.

## Electronic supplementary material

Below is the link to the electronic supplementary material.

Supplementary Material 1

Supplementary Material 2

## Data Availability

An earlier study by Lee et al. provided the main data used in this investigation. The database file of the manuscript was 10.1371/journal.pone.0221400.

## Abbreviations

TyG-BMI: Triglyceride glucose-body mass index
GDM: Gestational diabetes mellitus
BMI: Body mass index
AST: Aspartate aminotransferase
ALT: Alanine aminotransferase
GGT: Gamma-glutamyl transferase
TC: Total cholesterol
TG: Triglyceride
HDL: High-density lipoprotein cholesterol
LDL: Low-density lipoprotein cholesterol
NAFLD: Nonalcoholic fatty liver disease
FPG: Fasting plasma glucose
IR: Insulin resistance
HOMA-IR: Homeostasis model assessment-insulin resistance
ROC: Receiver operating characteristic
AUC: Area under the curve
OR: Odds ratio
CI: Confidence interval
