# Supplementary material for: Association between triglyceride glucose-body mass index and gestational diabetes mellitus: a prospective cohort study
Source: BMC Pregnancy Childbirth. 2025 Feb 17;25:170. doi: 10.1186/s12884-025-07294-9 (PMC11834603; doi:10.1186/s12884-025-07294-9)
Supplement: Supplementary file 1 — Supplementary Material 1 [file 12884_2025_7294_MOESM1_ESM.pdf]

YIMORE

EDITING CERTIFICATE

To whom it may concern:

This document is to confirm that *Zibo Yimore Translation CO. LTD* happily provided English proofreading services for the following thesis.

Author: Xiaomin Liang, Kai Lai, Xiaohong Li, Di Ren, Shuiqing Gui, Ying Li, Zemao Xing

Topic: Association between triglyceride glucose-body mass index and gestational diabetes mellitus: a prospective cohort study

We performed comprehensive language editing, including corrections in grammar, punctuation, and syntax to the best of our abilities. The revised document was returned to the author on 01/27/2025. We are not aware of any changes made to the manuscript after this date and have kept a copy of the original file for any potential legal dispute.

Sincerely,

Henry Graff

Zibo Yimore Translation CO. LTD

Tel: 0533-2775538 Email:yimorezhls@163.com
