# Supplementary material for: Association between triglyceride glucose-body mass index and gestational diabetes mellitus: a prospective cohort study
Source: BMC Pregnancy Childbirth. 2025 Feb 17;25:170. doi: 10.1186/s12884-025-07294-9 (PMC11834603; doi:10.1186/s12884-025-07294-9)
Supplement: Supplementary file 2 — Supplementary Material 2 [file 12884_2025_7294_MOESM2_ESM.docx]

**Association between triglyceride glucose-body mass index and gestational diabetes mellitus: a prospective cohort study**

Xiaomin Liang^1^, Kai Lai^1^, Xiaohong Li^1^, Di Ren^1^

Shuiqing Gui^1*^*^†^*, Ying Li^1*^*^†^,* Zemao Xing^1*^*^†^*

^1^Department of Critical Care Medicine, Shenzhen Second People’s Hospital, The First Affiliated Hospital of Shenzhen University, Shenzhen, China.

Supplementary Table 1 Collinearity check (VIF selection)

|  | Step 1 | Step 2 | Step 3 | Step 4 |
| --- | --- | --- | --- | --- |
| TyG-BMI | 149 | 1.9 | 1.9 | 1.8 |
| Age | 1.1 | 1.1 | 1.1 | 1.1 |
| AST | 1.4 | 1.4 | 1.4 | 1.4 |
| ALT | 1.7 | 1.7 | 1.7 | 1.7 |
| GGT | 1.3 | 1.3 | 1.3 | 1.3 |
| Insulin | 16.9 | 16.8 | 16.8 | 1.7 |
| Parity | 1.2 | 1.2 | 1.2 | 1.2 |
| BMI | 118.8 | NA | NA | NA |
| TC | 80.7 | 80.6 | NA | NA |
| TG | 19.6 | 11 | 1.4 | 1.3 |
| HDL | 19.3 | 19.3 | 1.1 | 1.1 |
| LDL | 54.1 | 54.1 | 1.1 | 1.1 |
| FPG | 2.9 | 1.7 | 1.7 | 1.4 |
| HOMA_IR | 17.5 | 17.4 | 17.4 | NA |
| Hepatic steatosis | 1.2 | 1.2 | 1.2 | 1.2 |

HOMA-IR and TC were excluded as confounding factors due to their collinearity (Supplementary Table 1).

Supplementary Table 2 Characteristics between included and excluded participants

| Characteristics | Included | Excluded | P-value |
| --- | --- | --- | --- |
| Participants | 588 | 35 |  |
| Age (years) | 32.07 ± 3.80 | 32.11 ± 4.90 | 0.943 |
| Pre-pregnancy BMI (kg/m^2^) | 22.02 ± 3.48 | 23.12 ± 3.65 | 0.074 |
| AST (IU/L) | 16.00 (14.00-20.00) | 18.00 (15.00-23.50) | 0.395 |
| ALT (IU/L) | 11.00 (8.00-15.00) | 14.00 (9.50-17.50) | 0.696 |
| GGT (IU/L) | 12.00 (10.00-15.00) | 13.00 (11.00-18.00) | 0.207 |
| TC (mg/dL) | 172.80 ± 27.21 | 185.67 ± 28.66 | 0.071 |
| TG (mg/dL) | 118.82 ± 47.49 | 144.73 ± 68.93 | 0.040 |
| HDL (mg/dL) | 64.90 ± 13.55 | 67.36 ± 15.09 | 0.489 |
| LDL (mg/dL) | 84.02 ± 21.81 | 89.36 ± 24.71 | 0.351 |
| FPG (mg/dL) | 77.01 ± 9.72 | 81.43 ± 6.39 | 0.091 |
| Insulin (μIU/mL) | 8.40 (5.40-11.55) | 9.70 (7.50-13.25) | 0.209 |
| HOMA-IR | 1.50 (1.00-2.30) | 1.95 (1.50-2.55) | 0.606 |
| Parity |  |  | 0.265 |
| No | 309 (52.55%) | 15 (42.86%) |  |
| Yes | 279 (47.45%) | 20 (57.14%) |  |
| GDM |  |  | 0.157 |
| No | 552 (93.88%) | 19 (86.36%) |  |
| Yes | 36 (6.12%) | 3 (13.64%) |  |
| Hepatic steatosis |  |  | 0.276 |
| Grade 0 | 478 (81.29%) | 27 (77.14%) |  |
| Grade 1 | 85 (14.46%) | 5 (14.29%) |  |
| Grade 2 | 17 (2.89%) | 3 (8.57%) |  |
| Grade 3 | 8 (1.36%) | 0 (0.00%) |  |

Values are reported as mean± SD, median (Q1-Q3), or N (%).
